# Sleep quality and sex-related factors in adult patients with immune-mediated diabetes: a large cross-sectional study

**DOI:** 10.1007/s00592-023-02036-9

**Published:** 2023-02-17

**Authors:** Claudio Bongiorno, Simona Moscatiello, Michele Baldari, Enrico Saudelli, Stefano Zucchini, Giulio Maltoni, Danilo Ribichini, Alessia Bruco, Valentina Lo Preiato, Gilberto Laffi, Uberto Pagotto, Guido Di Dalmazi

**Affiliations:** 1 Division of Endocrinology and Diabetes Prevention and Care, IRCCS Azienda Ospedaliero-Universitaria Di Bologna, Bologna, Italy; 2 Department of Medical and Surgical Sciences (DIMEC), Alma Mater Studiorum University of Bologna, Bologna, Italy; 3 Pediatric Unit,, IRCCS Azienda Ospedaliero-Universitaria Di Bologna, Bologna, Italy

**Keywords:** Autoimmune diabetes, Type 1 diabetes, Sleep, Sleep quality, Pittsburgh Sleep Quality Index, Sex

## Abstract

**Aim:**

To analyze sleep quality and its relationships with clinical and biochemical features in a large cohort of adults with autoimmune diabetes.

**Methods:**

We administered to 553 patients with autoimmune diabetes the questionnaires: Pittsburgh Sleep Quality Index (PSQI), diabetes distress scale, diabetes-related quality of life and diabetes treatment satisfaction questionnaire. We excluded patients with missing HbA1c ± 4 months from PSQI administration or incorrect PSQI compilation (*n* = 110).

**Results:**

Altered sleep quality was recorded in 142/443 subjects (32%), insufficient total sleep time in 177/443 (40%). The altered sleep quality group had higher HbA1c (median 56 mmol/mol [interquartile range-IQR 49–62] *vs* 59 [IQR 52–68]; *P* < 0.001), higher average HbA1c in the previous 36 months (59 mmol/mol [IQR 54–68] vs 56 [IQR 51–62]; *P* < 0.001), and more individuals with HbA1c > 53 mmol/mol (74.6% *vs* 62.8%; *P* = 0.014). Diabetes duration (*P* = 0.63), type of insulin delivery (*P* = 0.48) and glucose monitoring (*P* = 0.35) were uninfluential. Patients with altered sleep quality showed higher prevalence of autoimmune (42 *vs* 28%; *P* = 0.005) and mental diseases (12 *vs* 4%; *P* = 0.002); there were greater emotional distress, and lower quality of life and treatment satisfaction (*P* < 0.001 for all), irrespective of sex. Men with altered sleep quality had higher HbA1c and prevalence of autoimmune diseases. Women showed greater prevalence of psychiatric disorders. Average HbA1c of the previous 36 months, autoimmune or psychiatric disorders were independent predictive factors for altered sleep quality.

**Conclusion:**

One-third of the patients with autoimmune diabetes showed altered sleep quality, which associates with worse glycemic control, and autoimmune and mental disorders, with sex-specific differences.

**Supplementary Information:**

The online version contains supplementary material available at 10.1007/s00592-023-02036-9.

## Introduction

The proportion of patients with autoimmune diabetes mellitus (AD) achieving an acceptable glycemic control is modest [1] and, from a recent analysis of the United States registry, it is lowering further [2]. Compliance to therapy and lifestyle interventions are of paramount importance as the development of AD complications is tightly linked to elevated glucose exposure [1]. Obtaining and maintaining good sleep quality greatly influences immune regulation and metabolic, neurocognitive, and mental health [3]; indeed, it is slowly gaining space in the clinical setting: the first authoritative recommendation of its evaluation dates to 2017 ADA Standards of Medical Care [4], with an initial focus on type 2 diabetes (T2D) which has remained unchanged to date [5]. Additionally, the lack of specific indications on sleep quality assessment in AD might have led to the relative paucity of literature in T1D and Latent Autoimmune Diabetes of the Adult (LADA), compared to T2D [6]. A few previous studies have analyzed the sleep quality of patients with AD, however, with a fair heterogeneity in the populations, study designs, and methods of sleep assessment, and reporting a wide prevalence range of altered sleep quality (26–67%) and heterogeneous associated factors [3, 6, 7]. Therefore, this study aims to investigate the sleep quality in a large cohort of AD patients, and to analyze potential associated factors.

## Methods

### Patients

During the SARS-CoV-2 pandemic, the Endocrinology and Diabetes Prevention and Care Unit of the IRCCS Azienda Ospedaliero-Universitaria of Bologna aided with the vaccine administration campaign, administering the first and second dose to the patients deemed to be more fragile, and among them the patients with diabetes.

We offered the study participation to all patients with autoimmune diabetes (either T1D and LADA) under insulin treatment receiving the second dose of the SARS-CoV-2 vaccine between April 14, 2021 and May 14, 2021.

### Sleep assessment and questionnaires

Perceived sleep quality over the previous month was assessed using the validated Italian Pittsburgh Sleep Quality Index (PSQI) questionnaire [9]. PSQI score is calculated from 19 self-rated questions, which are distributed in seven components each evaluated on a 0–3 scale, with 0 being the most and 3 being the least favorable. The components assess subjective sleep quality, latency and duration, habitual sleep efficiency, sleep disturbances, sleeping medications, daytime dysfunction. Component scores are summed to obtain a global PSQI score ranging from 0 to 21, with values > 5 being indicative of poor sleep. In addition, the sleep duration component was used to identify subjects with a reduced sleep duration using as a cut-off a perceived duration < 7 h [10, 11].

We also administered the diabetes distress scale (DDS), the diabetes-related quality of life (DQOL) and the diabetes treatment satisfaction questionnaire (DTSQ).

The DDS measures the diabetes-related emotional distress through 17 questions, self-rated on a 6-point Likert scale, belonging to four subscales each investigating a different distress-related domain: emotional burden, physician-related distress, regimen-related distress, and diabetes-related interpersonal distress [12]. Scores between 2.0–2.9 and ≥ 3.0 are associated, respectively, to moderate and high distress [13]. We have chosen the 2.0 cut-off to define distressed patients.

The DQOL, developed specifically for assessing the quality of life in persons with diabetes on insulin therapy, uses a 5-point Likert scale for the 46 self-rated items, scored from 1 (best outcome) to 5 (worst outcome) [14]. The items assess different determinants of the quality of life, grouped into four primary subscales: satisfaction, impact, diabetes worry, social/vocational worry. Lower values are indicative of better quality of life.

The DTSQ estimates satisfaction toward diabetes treatment and is suitable both for evaluating variations after treatment modification and for comparisons among subjects with different treatments [15]. Each of the 8 items is rated from 6 (best outcome) to 1 (worst outcome), the score is proportional to the satisfaction.

### Clinical, biochemical, and continuous glucose monitoring (CGM) data

We retrieved clinical and biochemical data, data on the treatment regimen and glucose monitoring in a time span of ± 4 months from the questionnaires’ administration. Poorly controlled diabetes was defined by HbA1c > 7.0% (53 mmol/mol). We additionally calculated the mean and the standard deviation of all HbA1c measurements over the past 3 years (mean follow-up time 29.9 months, range 3.0–35.9) in all patients having at least two HbA1c measurements at intervals of 6 ± 4 months (*n* = 435, 8 patients excluded).

We remotely retrieved, and included in the analysis, CGM data during the last 14 days from patients achieving a sensor use of > 70% (*n* = 91); CGM metrics were calculated as previously described [16].

### Selection of the patients

We enrolled 553 subjects with autoimmune diabetes. After excluding 44 patients without clinical data, 22 patients without HbA1c measurement, and 44 patients with incomplete/incorrect PSQI questionnaire completion, the analysis was performed on a final cohort of 443 patients (Supplementary Fig. 1).

### Statistical analysis

Data are shown as median and interquartile range (IQR) if not otherwise specified, or as frequencies. Continuous variables were analyzed by independent-samples *T* test or independent-samples Mann–Whitney *U* test, and categorical variables were investigated by *χ*^2^ test. Multinomial logistic regression model with a backward stepwise elimination was built to analyze the independent predictors associated with altered sleep quality. Sex, age, type of insulin administration (MDI *vs* CSII), and glucose monitoring (SMBG *vs* CGM and subtypes), autoimmune or psychiatric disorders, HbA1c at the time of the questionnaire compilation and mean HbA1c during the previous 3 years were used as predictors. The model was built using the following criteria for backwards stepwise elimination: 100 maximum iterations, 5 maximum step-halving, 10^–6^ parameter convergence, delta of 0, 10^–8^ as singularity tolerance. As for the stepwise options, the entry probability was 0.05, with a removal probability of 0.1 and the likelihood ratio as entry/removal test. Odds ratios (ORs) were computed together with their 95% confidence interval (CI). Statistical analysis was performed using SPSS V.26. P values < 0.05 were considered significant.

## Results

Anthropometric, clinical, and biochemical characterization of the whole study population is shown in Supplementary Table 1. Median age was 49 years (IQR 33–59) and 47% of the patients were females. Most subjects were affected by T1D (93.5%), and the median disease duration was 22.2 years (IQR 13.1–31.3). The majority of the patients were under MDI (83.5%) and 66.1% of them were using a CGM device.

**Fig. 1:**
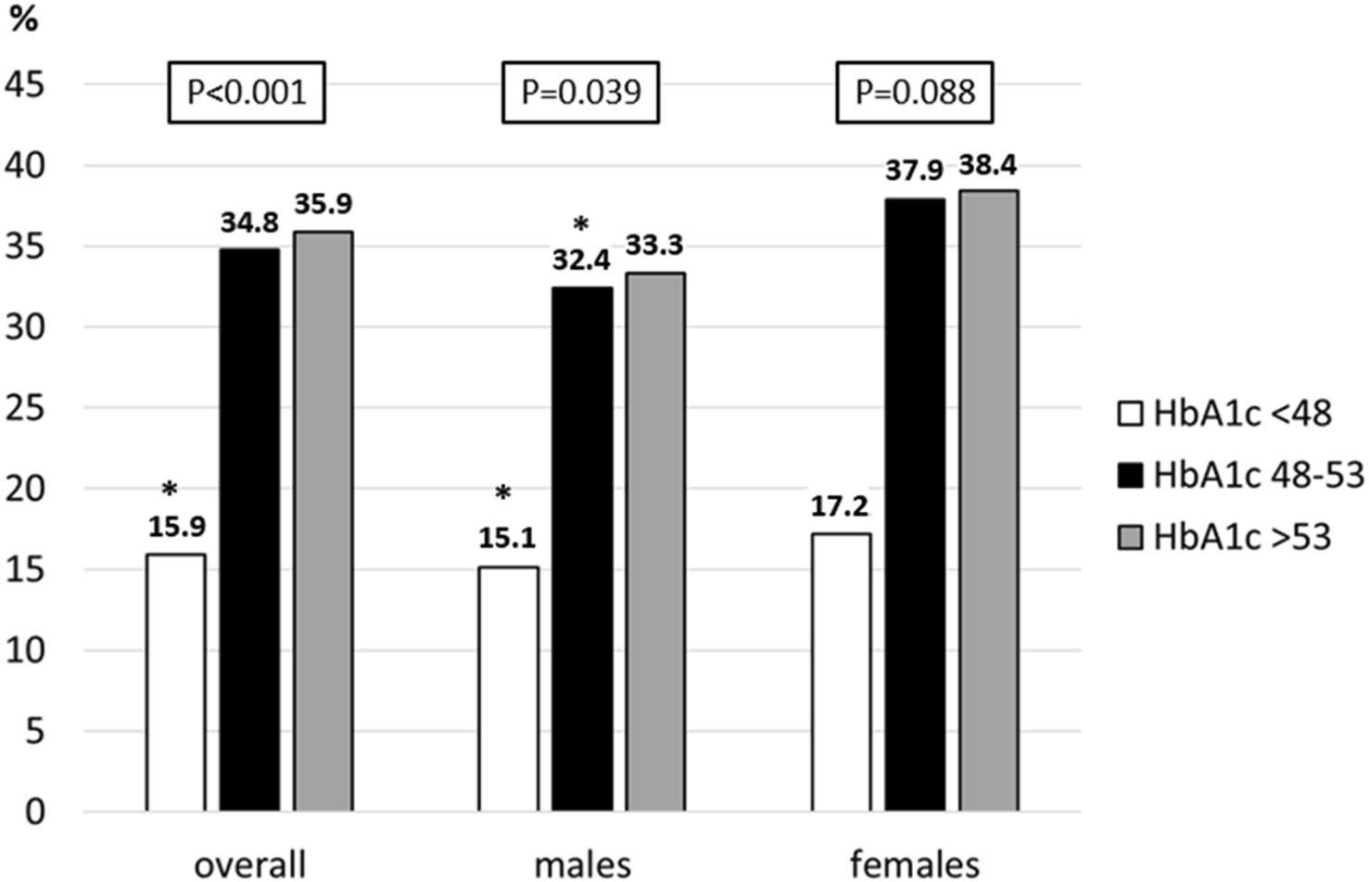
Proportion of patients with altered Pittsburgh Sleep Quality Index (> 5) among categories of HbA1c, overall and stratified by sex. Categories: < 6.5% (48 mmol/mol), 6.5–7.0% (48–53 mmol/mol), > 7% (53 mmol/mol). *P < 0.05 vs all

Altered sleep quality detected by PSQI questionnaire was found in 142 subjects (32.1%, from now on referred as “poor sleepers”, in contrast to “normal sleepers”), while reduced total sleep time (TST) was observed in 177 patients (40.0%, “short sleepers”, in contrast to “normal sleepers”) (Supplementary Table 1). The characteristics of the population stratified by PSQI results are shown in Table 1. No differences in anthropometric characteristics, as well as type of glucose monitoring and insulin administration were found between poor and normal sleepers. HbA1c was higher in the altered sleep quality group (7.5 [IQR 6.9–8.0] *vs* 7.3 [IQR 6.6–7.8] %; 58 [IQR 52–64] *vs* 56 [IQR 49–62] mmol/mol; *P* < 0.001). When stratified by HbA1c categories, the proportion of poor sleepers was 13/82 (15.9%), 23/66 (34.8%), and 106/295 (35.9%) in patients with HbA1c < 6.5% (48 mmol/mol), 6.5–7.0% (48–53 mmol/mol), and ≥ 7.0% (53 mmol/mol), respectively (*P* < 0.001), as shown in Fig. 1. Notably, no significant difference in the prevalence of altered sleep quality was found between patients with HbA1c 6.5–7.0% (48–53 mmol/mol) and ≥ 7.0% (53 mmol/mol).

**Table 1:** Population characteristics stratified by Pittsburgh Sleep Quality Index results

		Normal sleepers (*n* = 301)	Poor sleepers (*n* = 142)	*P* value
Demographic and anthropometric characteristics				
	Age, years	47.0 (34.0–57.0)	51.0 (32.8–60.0)	0.258
	Sex: Female, *n* (%)	135 (44.9)	74 (52.1)	0.153
	Body Mass Index, Kg/m^2^	24.0 (22.0–26.7)	24.6 (22.2–27.4)	0.405
	Smoker status			
	Non-smokers, *n* (%)	178 (59.1)	72 (50.7)	0.247
	Actively smoking, *n* (%)	64 (21.3)	36 (25.4)	
	Previously smoking, *n* (%)	59 (19.6)	34 (23.9)	
*Diabetes-related characteristics*				
	Diabetes Duration, years	22.7 (12.4–32.3)	21.2 (13.4–29.5)	0.627
	Diagnosis			
	T1D, *n* (%)	284 (94.4)	130 (91.5)	0.266
	LADA, *n* (%)	17 (5.6)	12 (8.5)	
	TDD/Kg Ratio, U/(day ⋅ Kg)	0.53 (0.4–0.7)	0.58 (0.4–0.7)	0.061
	Insulin administration			
	MDI, *n* (%)	254 (84.4)	116 (81.7)	0.475
	CSII, *n* (%)	47 (15.6)	26 (18.3)	
	Glucose monitoring: CGM	114 (68.7)	42 (61.8)	0.360
	Glucose monitoring			
	SMPG, *n* (%)	103 (34.2)	47 (33.1)	0.354
	isCGM, *n* (%)	149 (49.5)	64 (45.1)	
	rtCGM, *n* (%)	49 (16.3)	31 (21.8)	
	Insulin regimen in MDI treatment			
	Basal only, *n* (%)	8 (3.1)	5 (4.3)	0.574
	Basal bolus, *n* (%)	246 (96.9)	111 (95.7)	
	HbA1c, %	7.3 (6.6–7.8)	7.5 (6.9–8.4)	< 0.001
	HbA1c, mmol/mol	56 (49–62)	59 (52–68)	< 0.001
	36-months average HbA1c, % †	7.3 (6.8–7.8)	7.5 (7.1–8.4)	< 0.001
	36-months average HbA1c, mmol/mol†	56 (51–62)	59 (54–68)	< 0.001
	36-months SD of average HbA1c, mmol/mol†	4.2 (2.9–5.8)	4.3 (3.0–6.6)	0.368
*Associated diseases and complications*				
	Psychiatric disease, *n* (%)	12 (4.0)	17 (12.0)	0.002
	History of cancer, *n* (%)	12 (4.0)	5 (3.5)	0.812
	Thyroid disease, *n* (%)	86 (28.6)	51 (35.9)	0.119
	Autoimmune thyroiditis, *n* (%)	67 (22.3)	43 (30.3)	0.068
	Celiac disease, *n* (%)	13 (4.3%)	15 (10.6)	0.012
	Patients with at least one additional autoimmune disorder, *n* (%)	85 (28.2)	59 (41.5)	0.005
	Hypertension, *n* (%)	66 (21.9)	36 (25.4)	0.424
	Cardiovascular diseases, *n* (%)	10 (3.3)	9 (6.3)	0.144
	Diabetic retinopathy, *n* (%)	96 (31.9)	45 (31.7)	0.966
	Diabetic nephropathy, *n* (%)	6 (2.0)	6 (4.2)	0.177
*Diabetes Distress Scale (n = 420)*				
	Population, *n*	287	133	
	Global score	1.59 (1.24–2.24)	2.12 (1.5–3.3)	< 0.001
	Emotional burden score	1.8 (1.4–2.8)	2.6 (1.8–3.8)	< 0.001
	Physician-related distress score	1.25 (1.0–2.0)	1.25 (1.0–2.63)	0.052
	Regimen-related distress score	1.6 (1.2–2.2)	2.0 (1.5–3.4)	< 0.001
	Diabetes-related interpersonal distress score	1.33 (1.0–2.0)	2.0 (1.0–3.0)	< 0.001
	Patients with altered score			
	Emotional burden, *n* (%)	119 (41.5)	83 (62.4)	< 0.001
	Physician-related distress**, ***n* (%)	63 (22.0)	44 (33.1)	0.015
	Regimen-related distress**, ***n* (%)	74 (25.8)	64 (48.1)	< 0.001
	Diabetes-related interpersonal distress**, ***n* (%)	54 (18.8)	53 (39.8)	< 0.001
*Diabetes-related Quality of Life (n = 305)*				
	Population, *n*	216	89	
	Score	1.67 (1.51–1.87)	1.98 (1.72–2.67)	< 0.001
*Diabetes Treatment Satisfaction Questionnaire status version (n = 416)*				
	Population, *n*	282	134	
	Score	30.0 (26.0–34.0)	29.0 (24.0–32.0)	< 0.001

Data are expressed as median with interquartile range in parentheses, or as frequencies

T1D, type 1 diabetes; LADA, latent autoimmune diabetes of the adult; TDD, total daily (insulin) dose; MDI, multiple daily injections; CSII, continuous subcutaneous insulin infusion; SMPG, self-monitoring of plasma glucose; isCGM, intermittently scanned continuous glucose monitoring (device); rtCGM, real-time continuous glucose monitoring (device); HbA1c, glycated hemoglobin; SD, standard deviation

^†^ *n* = 435

Average HbA1c in the previous 36 months before PSQI administration was significantly higher in poor than normal sleepers (7.5 [IQR 7.1–8.4] *vs* 7.3 [IQR 6.8–7.8]; 59 mmol/mol [IQR 54–68] *vs* 56 mmol/mol [IQR 51–62]; *P* < 0.001), whereas standard deviation of the average HbA1c was not (4.3 [IQR 3.0–6.6] *vs* 4.2 [IQR 2.9–5.8] mmol/mol; *P* = 0.368).

Patients with altered sleep quality showed a higher prevalence of psychiatric disorders (12.0% *vs* 4.0%; *P* = 0.002) and autoimmune diseases (41.5% *vs* 28.2%; *P* < 0.005), especially celiac disease (10.6% *vs* 4.3%; *P* = 0.012). No difference in the prevalence of other comorbidities or complications of diabetes was found between groups.

When analyzed according to the TST, short sleepers had higher HbA1c than normal sleepers (7.5 [IQR 6.9–8.1] *vs* 7.3 [IQR 6.6–7.8] %; 58 mmol/mol [IQR 52–65] *vs* 56 mmol/mol [IQR 49–62]; *P* = 0.008), without differences in the remaining parameters (Supplementary Table 2). Only 3 patients (0.7%) reported a TST greater than 9 h [10, 11], thus no specific analysis on this subgroup was performed.

The analysis of the results of DDS, DRQoL, and DTSQ in poor and normal sleepers is shown in Table 1. The group with altered sleep quality suffered from a higher diabetes-related emotional distress, evaluated by comparing both the DDS global score value (*p* < 0.001) and the number of patients with an altered global score (*p* < 0.001). Diabetes-related quality of life and diabetes treatment satisfaction were significantly greater in normal versus poor sleeper groups (*p* < 0.001 for both).

When analyzed separately by sex (Table 2), we confirmed that males with poor sleep quality had higher HbA1c (*P* = 0.001) and a larger prevalence of autoimmune diseases (*P* = 0.012), particularly celiac disease (*P* = 0.003), than normal sleepers. An additional significant difference was a higher TDD/Kg in poor versus normal male sleepers (*p* = 0.026). Conversely, women reporting poor sleep quality had a higher prevalence of psychiatric disorders than normal sleepers (*P* = 0.016), in the absence of other relevant differences. Slightly higher values of average HbA1c in the previous 3 years were identified in poor sleepers than normal sleepers, however with *P* values close to significance, questioning about the relevance of this difference. The analysis of the results of DDS, DRQoL, and DTSQ in poor and normal sleepers divided by sex confirmed the results obtained in the whole cohort (Supplementary Table 3).

**Table 2:** Population characteristics stratified by sex and Pittsburgh Sleep Quality Index results

		Males			Females		
		Normal sleepers (*n* = 166)	Poor sleepers (*n* = 68)	*P* value	Normal sleepers (*n* = 135)	Poor sleepers (*n* = 74)	*P* value
*Demographic and anthropometric characteristics*							
	Age, years	48 (35–57)	50 (33–59)	0.646	46 (33–59)	52 (30–61)	0.274
	Body Mass Index, Kg/m^2^	24.7 (22.7–26.8)	25.2 (22.8–27.4)	0.665	23.0 (21.3–26.5)	23.6 (21.6–28.2)	0.307
	Smoker status						
	Non-smokers, *n* (%)	90 (54.2)	30 (44.1)	0.266	88 (65.2)	42 (56.8)	0.367
	Actively smoking, *n* (%)	40 (24.1)	17 (25.0)		24 (17.8)	19 (25.7)	
	Previously smoking, *n* (%)	36 (21.7)	21 (30.9)		23 (17.0)	13 (17.6)	
*Diabetes-related characteristics*							
	Diabetes Duration, years	22.7 (12.3–32.3)	18.0 (13.4–28.3)	0.358	22.7 (12.4–31.4)	22.2 (14.0–30.6)	0.807
	Diagnosis						
	T1D, *n* (%)	157 (94.6)	62 (91.2)	0.335	127 (94.1)	68 (91.9)	0.546
	LADA, *n* (%)	9 (5.4)	6 (8.8)		8 (5.9)	6 (8.1)	
	TDD/Kg Ratio, U/(day ⋅ Kg)	0.55 (0.43–0.67)	0.61 (0.49–0.72)	0.026	0.51 (0.41–0.64)	0.53 (0.36–0.70)	0.441
	Insulin administration						
	MDI, *n* (%)	139 (83.7)	57 (83.8)	0.987	115 (85.2)	59 (79.7)	0.336
	CSII, *n* (%)	27 (16.3)	11 (16.2)		20 (14.8)	15 (20.3)	
	Glucose monitoring: CGM	114 (68.7)	42 (61.8)	0.309	84 (62.2)	53 (71.6)	0.171
	Glucose monitoring: device class						
	SMPG, *n* (%)	52 (31.3)	26 (38.2)	0.239	51 (37.8)	21 (28.4)	0.312
	isCGM, *n* (%)	86 (51.8)	27 (39.7)		63 (46.7)	37 (50.0)	
	rtCGM, *n* (%)	28 (16.9)	15 (22.1)		21 (15.6)	16 (21.6)	
	Insulin regimen in MDI treatment						
	Basal only, *n* (%)	4 (2.9)	2 (3.5)	0.816	4 (3.5)	3 (5.1)	0.610
	Basal bolus, *n* (%)	135 (97.1)	55 (96.5)		111 (96.5)	56 (94.9)	
	HbA1c, %*	7.1 (6.5–7.6)	7.5 (6.9–8.2)	0.001	7.5 (6.7–8.1)	7.5 (7.2–8.4)	0.147
	HbA1c, mmol/mol*	54 (47–60)	59 (52–66)		58 (50–65)	59 (55–68)	
	36-months average HbA1c, %†	7.2 (6.7–7.7)	7.5 (7.0–8.2)	0.005	7.5 (7.0–8.1)	7.7 (7.2–8.5)	0.049
	36-months average HbA1c, mmol/mol†	55 (50–61)	58 (53–66)		58 (53–65)	61 (55–69)	
	36-months SD of average HbA1c, mmol/mol†	4.6 (3.2–6.1)	4.3 (3.2–6.7)	0.799	5.0 (3.8–6.8)	5.5 (4.0–7.7)	0.282
*Associated diseases and complications*							
	Psychiatric disease, *n* (%)	4 (2.4)	5 (7.4)	0.074	8 (5.9)	12 (16.2)	0.016
	History of cancer, *n* (%)	4 (2.4)	2 (2.9)	0.815	8 (5.9)	3 (4.1)	0.562
	Thyroid disease, *n* (%)	25 (15.1)	11 (16.2)	0.830	61 (45.2)	40 (54.1)	0.220
	Autoimmune thyroiditis, *n* (%)	18 (10.8)	10 (14.7)	0.408	49 (36.3)	33 (44.6)	0.240
	Celiac disease, *n* (%)	4 (2.4)	8 (11.8)	0.003	9 (6.7)	7 (9.5)	0.468
	Patients with at least one additional autoimmune disorder, *n* (%)	29 (17.5)	22 (32.4)	0.012	56 (41.5)	37 (50.0)	0.236
	Hypertension, *n* (%)	43 (25.9)	19 (27.9)	0.748	23 (17.0)	17 (23.0)	0.297
	Cardiovascular diseases, *n* (%)	4 (2.4)	5 (7.4)	0.074	6 (4.4)	4 (5.4)	0.756
	Diabetic retinopathy, *n* (%)	51 (30.7)	23 (33.8)	0.643	45 (33.3)	22 (29.7)	0.593
	Diabetic nephropathy, *n* (%)	5 (3.0)	4 (5.9)	0.300	1 (0.7)	2 (2.7)	0.254

Data are expressed as median with interquartile range in parentheses, or as frequencies

T1D, type 1 diabetes; LADA, latent autoimmune diabetes of the adult; TDD, total daily (insulin) dose; MDI, multiple daily injections; CSII, continuous subcutaneous insulin infusion; SMPG, self-monitoring of plasma glucose; isCGM, intermittently scanned continuous glucose monitoring (device); rtCGM, real-time continuous glucose monitoring (device); HbA1c, glycated hemoglobin

^*^ Male normal sleepers vs other groups (male poor sleepers, female normal sleeper, female poor sleeper): *P* < 0.001 for all comparisons

^†^ *n* = 435

Next, we proceeded to find potential independent factors associated with altered sleep quality using a multinomial logistic regression model (Table 3). Mean HbA1c during the previous 3 years (OR 1.03 per 1 mmol/mol increase, 95% CI 1.01–1.05; *p* = 0.001), presence of autoimmune disorders (OR 1.82, 95% CI 1.18–2.80; *P* = 0.007) or psychiatric diseases (OR 3.15, 95% CI 1.43–6.95; *P* = 0.004) were among independent potential predictive factors for altered sleep quality.

**Table 3:** Results of the logistic regression analysis of the possible independent predictors associated with altered sleep quality

Significant predictors in the model		Odds Ratio (95% confidence interval)	*P*-value
	Autoimmune disorders (presence vs absence)	1.82 (1.18–2.80)	0.007
	Psychiatric conditions (presence vs absence)	3.15 (1.43–6.95)	0.004
	Three-years mean HbA1c (1 mmol/mol increase)	1.03 (1.01–1.05)	0.001

The following parameters were excluded by the backward stepwise procedure: HbA1c (1 mmol/mol increase) (*P* = 0.976), glucose measurement device (SMPG vs CGM) (*P* = 0.838), sex (females vs males) (*P* = 0.809), age (1 year increase) (0.415), insulin administration device (MDI vs CSII) (*P* = 0.507)

We finally performed the analysis of the 14-days CGM metrics for the subjects with available data (*n* = 96). No statistically significant difference was observed between poor and normal sleepers (Supplementary Table 4).

## Discussion

In our cohort of patients with AD we found a relevant proportion of altered sleep quality (32.1%) and reduced sleep duration (40.0%), representing the largest study published so far on young and middle-aged adults. Additionally, for the first time, we found different parameters associated with sleep quality according to sex.

Multiple factors can alter sleep architecture, duration, or quality, especially in the context of AD, considering the role of nocturnal hypoglycemia and its self-management behaviors, discomfort or anxiety related to fear of hypoglycemia, and possible polyuria; even AD complications can have a negative impact on sleep, such as neuropathic pain [3, 7].

Previous studies attempted to analyze the sleep quality in patients with AD by means of objective (PSG, actigraphy) and subjective measures (self-reported questionnaires), showing that patients with T1D had a worse sleep quality than control subjects only when assessed by questionnaires rather than PSG or actigraphy [6]. The method of assessment of sleep quality may explain those differences, since objective measures report the results of a single night, whereas questionnaires refer to self-reported quality of sleep during the previous month. The few studies investigating sleep quality through PSQI in adults with T1D have been conducted with a cross-sectional or retrospective design, on average on a non-obese population comprising a broad spectrum of ages (range of mean age 27–67 years) and glycemic control (range of mean HbA1c 7.2–8.3%; 55–67 mmol/mol), reporting a large heterogeneous prevalence of altered sleep quality, ranging from 27 to 67% (mean pooled prevalence 43.9%–CI 32.5–55.5) [17–25]. The largest prevalence of altered sleep quality in patients with T1D (67%) was reported by Gilsanz and colleagues and doubled that of our population. However, it must be acknowledged that only elderly patients were enrolled in that study (> 60 years old) and that no data on metabolic control of diabetes were reported. It is well known that sleep quality may worsen with increasing age [26], though in our cohort of individuals with autoimmune diabetes age did not appear to be a predictive factor. Additionally, the study of Suteau and colleagues, reporting a prevalence of 60%, involved patients with more diabetes-related complications than ours (46% patients with microvascular and 10% with macrovascular complications) and poorly controlled diabetes (mean HbA1c 7.6%; 60 mmol/mol), explaining the differences with our findings. In the remaining studies including patients with mean age like that of our patients, the prevalence of altered sleep quality was comparable to our results [18, 20].

Regarding the comparison with sleep data in the general population, no similar data in terms of population age and observation period are reported. Our data were collected in a very narrow time span, at variance from other studies which collected sleep data over longer periods and under variable conditions, due to the evolution of the different measures adopted by the single governments during the Covid19 pandemic. Furthermore, reported prevalence of sleep disorders is lacking, having most of the studies reported only the mean values of the PSQI score. Acknowledging the limitations of the following analysis, we tried to compare our data with those of the general population before Covid19 pandemic, derived from a large population-based study [27], stratifying the PSQI results by age and sex. Our cohort had generally higher PSQI values than the general population in both sexes, especially in young adults. Indeed, in subjects ≤ 39 years old, males had a score (mean ± SD) of 4.54 ± 2.21 and females of 4.86 ± 2.99 in our cohort, whereas in the general population was reported a PSQI score of 3.86 ± 2.69 in males and 4.21 ± 2.76 in females.

Overall, we showed a relationship between sleep quality and several patients’ characteristics. As also highlighted by the logistic regression analysis, long-term metabolic control of diabetes, autoimmune and psychiatric disorders were independent predictive factors of altered sleep quality in patients with AD. Additionally, we identified that those characteristics were different according to sex.

The relationship between sleep quality and diabetes metabolic control has been seldomly investigated. Only five studies reported the analysis of HbA1c levels by categories of sleep quality assessed by PSQI, showing no difference between poor and normal sleepers [17, 18, 20, 21, 24]. However, two additional studies assessing self-reported sleep quality by a non-specific questionnaire (PHQ9) reported higher HbA1c values in patients with poor sleep quality [28, 29], whereas studies using PSG or actigraphy did not [6]. At variance with other studies using PSQI, we showed that poor sleepers had greater HbA1c than normal sleepers; extending this finding, we showed that the proportion of patients with poor sleep quality was higher among HbA1c categories, with significant differences between values lesser or greater than 6.5% (48 mmol/mol). Additionally, the higher HbA1c levels in poor sleepers were confirmed even when including all the measurements of the previous 3 years, without difference in variability of HbA1c, allowing us to speculate that subjects with altered sleep quality may also have chronically high glucose levels. The differential impact of suboptimal glycemic control on sleep quality in males and females is novel. Males reporting normal sleep had significantly better glycemic control than males with poor sleep quality and females with or without poor sleep quality, suggesting a tighter relationship between glycemic control and sleep in males, contrary to females, whose sleep quality seems to be relatively independent of glycemic control. Our results are coherent also with the hypothesis that decreased insulin sensitivity may be a mechanism of altered sleep quality and duration [23], as highlighted by the increased TDD/Kg (a sign of insulin resistance) in males with poor sleep quality. In fact, insulin resistance has been reported to be influenced by sex and sex hormones levels, though the exact mechanism has not been discovered [30].

The analysis performed separately in males and females confirmed the higher prevalence of psychiatric disorders in patients with altered sleep quality only among females, whereas the larger prevalence of celiac and autoimmune diseases was identified only in men with low quality of sleep. Previous studies showed that patients with celiac disease may have a variable proportion of altered sleep quality; additionally, males showed a worse adherence to gluten-free diet than females [31] and patients non-adherent to gluten-free diet have more frequently T1D [32]. Recent data also highlighted that gluten-free diet may improve sleep quality [33]. Therefore, the relationship between sleep quality and celiac disease in male patients with T1D may be interpreted in this context, even though the impact of other autoimmune diseases should be considered. Indeed, the association between sleep and inflammation, which naturally is involved in autoimmune pathologies, is documented and bidirectional as the former modulates yet it is also modulated by the latter [34].

The altered sleep quality in our cohort was related to a significantly greater emotional burden, regimen-related and interpersonal distress caused by the pathology, in line with previous studies [35, 36], and lower satisfaction for diabetes treatment, eventually leading to a lower quality of life. Interestingly, those alterations were independent of sex. The presence of diabetes-related distress is associated with poor patient compliance, worse glycemic control and reduced psychological health [37]. The coexistence of low HbA1c, high quality of life and treatment satisfaction (with their demonstrated association) in people with good sleep quality might be another suggestion for a direct action of a suboptimal glycemic control on sleep. In addition, it remains to be established how and how much the poor sleep quality affects the distress in each of the highlighted subscales.

Lastly, relevant is the lack of association between sleep quality and the use of CGM devices or insulin pumps. However, it must be acknowledged our relatively low number of patients with available glucose data. In some studies, CGM technology has been associated with an overall improvement of subjective sleep quality, although the literature is heterogenous in terms of populations, devices, and methods. In addition, there is limited research on objective sleep data and in most of the device studies sleep quality was not the primary outcome, thus the analyses could have not been sufficiently powered to detect any changes. Studies on sensor augmented pumps provided mixed results in terms of effect on sleep [38].

The main limitations of the study are the cross-sectional design and the use of a self-reported method to evaluate sleep quality. Nonetheless, we chose the PSQI being it a more practical and faster tool to assess sleep quality, suitable for very large populations. In addition, questionnaires were collected during the SARS-CoV-2 pandemic, which may represent a confounding factor. However, we believe that this is a minor limitation since the prevalence of altered sleep quality in our patients was like that of previous studies. The strengths of our study are the large cohort involved (at the time of writing the largest in the literature focused on this age range), the inclusion of subjects with LADA, the analysis of the data separately by sex, and the completeness of clinical and biochemical data.

## Conclusions

A significant proportion of adult patients with autoimmune diabetes showed altered sleep quality, which was mostly related to poor glycemic control and the presence of autoimmune disorders in men, and psychological/psychiatric disorders in women. Sleep is a complex phenomenon influenced by multiple factors, which might not be equally relevant on an individual basis inside this specific but heterogeneous population. Addressing such factors, when possible, in a more targeted intervention could lead to better outcomes on sleep quality. Further studies are needed to address sex-related differences in the factors associated with altered sleep quality.

## Supplementary Information

Below is the link to the electronic supplementary material.

Supplementary file1 (DOCX 52 KB)

## References

[1] McKnight JA, Wild SH, Lamb MJE, Cooper MN, Jones TW, Davis EA (2015). Glycaemic control of Type 1 diabetes in clinical practice early in the 21st century: an international comparison. Diabet Med J Br Diabet Assoc.

[2] Foster NC, Beck RW, Miller KM, Clements MA, Rickels MR, DiMeglio LA (2019). State of type 1 diabetes management and outcomes from the t1d exchange in 2016–2018. Diabetes Technol Ther.

[3] Perez KM, Hamburger ER, Lyttle M, Williams R, Bergner E, Kahanda S, Cobry E, Jaser SS (2018). Sleep in type 1 diabetes: implications for glycemic control and diabetes management. Curr DiabRep.

[4] American Diabetes Association (2016). 3 comprehensive medical evaluation and assessment of comorbidities. Diabetes Care.

[5] American Diabetes Association Professional Practice Committee (2021). 4. Comprehensive medical evaluation and assessment of comorbidities: standards of medical care in diabetes—2022. Diabetes Care.

[6] Reutrakul S, Thakkinstian A, Anothaisintawee T, Chontong S, Borel A-L, Perfect MM (2016). Sleep characteristics in type 1 diabetes and associations with glycemic control: systematic review and meta-analysis. Sleep Med.

[7] Zhu B, Abu Irsheed GM, Martyn-Nemeth P, Reutrakul S (2021). Type 1 diabetes, sleep, and hypoglycemia. Curr Diab Rep.

[8] Perfect MM (2020). Sleep-related disorders in patients with type 1 diabetes mellitus: current insights. Nat Sci Sleep.

[9] Curcio G, Tempesta D, Scarlata S, Marzano C, Moroni F, Rossini PM (2013). Validity of the Italian version of the Pittsburgh Sleep Quality Index (PSQI). Neurol Sci.

[10] Consensus Conference Panel (2015). Recommended amount of sleep for a healthy adult: a joint consensus statement of the American academy of sleep medicine and sleep research society. Sleep.

[11] National Sleep Foundation (2020). How Much Sleep Do You Really Need? *Natl Sleep Found*. https://www.thensf.org/how-many-hours-of-sleep-do-you-really-need/. Accessed June 30, 2022

[12] Polonsky WH, Fisher L, Earles J, Dudl RJ, Lees J, Mullan J (2005). Assessing psychosocial distress in diabetes: development of the diabetes distress scale. Diabetes Care.

[13] Fisher L, Hessler DM, Polonsky WH, Mullan J (2012). When is diabetes distress clinically meaningful?: establishing cut points for the Diabetes Distress Scale. Diabetes Care.

[14] Labbrozzi D, Erle G, Meschi F (1996). La valutazione della qualità della vita nel paziente diabetico. Adattamento linguistico e validazione del Diabetes Quality-of- Life Questionnaire (DQOL). G Ital di Diabeto.

[15] Nicolucci A, Giorgino R, Cucinotta D, Zoppini G, Muggeo M, Squatrito S (2004). Validation of the Italian version of the WHO-Well-Being Questionnaire (WHO-WBQ) and the WHO-Diabetes Treatment Satisfaction Questionnaire (WHO-DTSQ). Diabetes Nutr Metab.

[16] Di Dalmazi G, Maltoni G, Bongiorno C, Tucci L, Di Natale V, Moscatiello S, Laffi G, Pession A, Zucchini S, Pagotto U (2020). Comparison of the effects of lockdown due to COVID-19 on glucose patterns among children, adolescents, and adults with type 1 diabetes: CGM study. BMJ Open Diabetes Res Care.

[17] Van Dijk M, Donga E, van Dijk JG, Lammers GJ, van Kralingen KW, Dekkers OM, Corssmit EP, Romijn JA (2011). Disturbed subjective sleep characteristics in adult patients with long-standing type 1 diabetes mellitus. Diabetologia.

[18] Nefs G, Donga E, Someren E, Bot M, Speight J, Pouwer F (2015). Subjective sleep impairment in adults with type 1 or type 2 diabetes: results from diabetes MILES–The Netherlands. Diabetes Res Clin Pract.

[19] Chontong S, Saetung S, Reutrakul S (2016). Higher sleep variability is associated with poorer glycaemic control in patients with type 1 diabetes. J Sleep Res.

[20] Denic-Roberts H, Costacou T, Orchard TJ (2016). Subjective sleep disturbances and glycemic control in adults with long-standing type 1 diabetes: The Pittsburgh’s Epidemiology of Diabetes Complications study. Diabetes Res Clin Pract.

[21] Martyn-Nemeth P, Phillips SA, Mihailescu D, Farabi SS, Park C, Lipton R (2018). Poor sleep quality is associated with nocturnal glycaemic variability and fear of hypoglycaemia in adults with type 1 diabetes. J Adv Nurs.

[22] Rusu A, Ciobanu D, Bala C, Cerghizan A, Roman G (2019). Social jetlag, sleep-related parameters, and glycemic control in adults with type 1 diabetes: Results of a cross-sectional study. J Diabetes.

[23] Mattos, A. C. M. T., Campos, Y. S., Fiorini, V. O., Sab, Y., Tavares, B. L., Velarde, L. G. C., Lima, G. A. B., & Cruz Filho, R. A. D. (2020). Relationship between sleep disturbances, lipid profile and insulin sensitivity in type 1 diabetic patients: a cross-sectional study. *Archives of endocrinology and metabolism; 64*(4):412–417. 10.20945/2359-3997000000228 10.20945/2359-3997000000228 PMC10522075 32267356

[24] Suteau, V., Saulnier, P. J., Wargny, M., Gonder-Frederick, L., Gand, E., Chaillous, L., Allix, I., Dubois, S., Bonnet, F., Leguerrier, A. M., Fradet, G., Delcourt Crespin, I., Kerlan, V., Gouet, D., Perlemoine, C., Ducluzeau, P. H., Pichelin, M., Ragot, S., Hadjadj, S., Cariou, B., … VARDIA study group (2020). Association between sleep disturbances, fear of hypoglycemia and psychological well-being in adults with type 1 diabetes mellitus, data from cross-sectional VARDIA study. *Diabetes research and clinical practice 160*:107988. 10.1016/j.diabres.2019.107988 10.1016/j.diabres.2019.107988 31866527

[25] Gilsanz P, Lacy ME, Beeri MS, Karter AJ, Eng CW, Whitmer RA (2020). Sleep quality and cognitive function in type 1 diabetes: findings from the study of longevity in diabetes (SOLID). Alzheimer Dis Assoc Disord.

[26] Li J, Vitiello MV, Gooneratne NS (2018). Sleep in normal aging. Sleep Med Clin.

[27] Hinz A, Glaesmer H, Brähler E, Löffler M, Engel C, Enzenbach C, Hegerl U, Sander C (2017). Sleep quality in the general population: psychometric properties of the Pittsburgh Sleep Quality Index, derived from a German community sample of 9284 people. Sleep Med.

[28] Bot M, Pouwer F, Jonge P, Tack CJ, Geelhoed-Duijvestijn PHLM, Snoek FJ (2013). Differential associations between depressive symptoms and glycaemic control in outpatients with diabetes. Diabet Med.

[29] Bächle C, Lange K, Stahl-Pehe A, Castillo K, Holl RW, Giani G (2015). Associations between HbA1c and depressive symptoms in young adults with early-onset type 1 diabetes. Psychoneuroendocrinology.

[30] Šimonienė D, Platūkiene A, Prakapienė E, Radzevičienė L, Veličkiene D (2020). Insulin resistance in type 1 diabetes mellitus and its association with patient’s micro- and macrovascular complications, sex hormones, and other clinical data. Diabetes Ther.

[31] Pasternack C, Hervonen K, Mansikka E, Reunala T, Kaukinen K, Salmi T (2022). Sex-differences in Gluten-free Dietary Adherence and Clinical Symptoms in Patients with Long-term Treated Dermatitis Herpetiformis. Acta Derm Venereol.

[32] Kivelä, L., Eurén, A., Repo, M., Huhtala, H., Kaukinen, K., & Kurppa, K. (2022). Coexisting Type 1 Diabetes, Persistent Symptoms, and Financial Issues Associate With Poorer Adherence to a Gluten-Free Diet in Celiac Disease After Transition From Pediatrics to Adult Care. *Front Nutr*. 10.3389/fnut.2022.883220 10.3389/fnut.2022.883220 PMC9200750 35719146

[33] Gamli IS, Basaran MK (2022). The effect of a gluten-free diet on sleep disturbances in children with celiac disease. NSS.

[34] Zielinski MR, Krueger JM (2011). Sleep and innate immunity. Front Biosci-Sch.

[35] Griggs S, Grey M, Ash GI, Li CR, Crawford SL, Hickman RL (2022). Objective sleep-wake characteristics are associated with diabetes symptoms in young adults with type 1 diabetes. Sci Diabetes Self-Manag Care.

[36] Fisher L, Hessler D, Polonsky W, Strycker L, Masharani U, Peters A (2016). Diabetes distress in adults with type 1 diabetes: prevalence, incidence and change over time. J Diabetes Complicat.

[37] Powers MA, Richter SA, Ackard DM, Craft C (2017). Diabetes distress among persons with type 1 diabetes: associations with disordered eating, depression, and other psychological health concerns. Diabetes Educ.

[38] Cobry EC, Karami AJ, Meltzer LJ (2022). Friend or foe: a narrative review of the impact of diabetes technology on sleep. Curr Diab Rep.

