# Supplementary material for: Sleep quality and sex-related factors in adult patients with immune-mediated diabetes: a large cross-sectional study
Source: Acta Diabetol. 2023 Feb 17;60(5):663–72. doi: 10.1007/s00592-023-02036-9 (PMC9936122; doi:10.1007/s00592-023-02036-9)
Supplement: Supplementary file 1 — Supplementary file1 (DOCX 52 KB) [file 592_2023_2036_MOESM1_ESM.docx]

**Supplementary material.**

Sleep quality and sex-related factors in adult patients with immune-mediated diabetes: a large cross-sectional study.

**Journal:** *Acta Diabetologica*.

**Authors**

Claudio Bongiorno^1,2^, Simona Moscatiello^1^, Michele Baldari^1^, Enrico Saudelli^1,2^, Stefano Zucchini^3^, Giulio Maltoni^3^, Danilo Ribichini^1^, Alessia Bruco^1,2^, Valentina Lo Preiato^1,2^, Gilberto Laffi^1^, Uberto Pagotto^1,2^, Guido Di Dalmazi^1,2^

**Affiliations**

^1^Division of Endocrinology and Diabetes Prevention and Care, IRCCS Azienda Ospedaliero-Universitaria di Bologna, Bologna, Italy. ^2^Department of Medical and Surgical Sciences (DIMEC), Alma Mater Studiorum University of Bologna, Bologna, Italy. ^3^Pediatric Unit, IRCCS Azienda Ospedaliero-Universitaria di Bologna, Bologna, Italy.

**Correspondence**

Guido Di Dalmazi, Division of Endocrinology and Diabetes Prevention and Care, Department of Medical and Surgical Sciences (DIMEC), Alma Mater Studiorum University of Bologna, IRCCS Azienda Ospedaliero-Universitaria di Bologna, Bologna, Italy. Tel: +39 051 2143009 Fax: +39 051 214 3080. Email: guido.didalmazi@unibo.it

**Supplementary Table 1. Characteristics of the whole population.**

| Demographic and anthropometric characteristics | | |
| --- | --- | --- |
|  | Population, n | 443 |
|  | Age, years | 49 (33.0-59.0) |
|  | Female, n (%) | 209 (47.2) |
|  | Body Mass Index, Kg/m^2^ | 24.2 (22.1–26.8) |
|  | Smoker status |  |
|  | Non-smokers, n (%) | 250 (56.4) |
|  | Actively smoking, n (%) | 100 (22.6) |
|  | Previously smoking, n (%) | 93 (21.0) |
| **Diabetes-related characteristics** | | |
|  | Diabetes Duration, years | 22.2 (13.1-31.3) |
|  | Diagnosis |  |
|  | T1D, n (%) | 414 (93.5) |
|  | LADA, n (%) | 29 (6.5) |
|  | TDD/Kg Ratio, U/(day ⋅ Kg) | 0.54 (0.42-0.68) |
|  | Insulin administration |  |
|  | MDI, n (%) | 370 (83.5) |
|  | CSII, n (%) | 73 (16.5) |
|  | Glucose monitoring |  |
|  | SMPG, n (%) | 150 (33.9) |
|  | isCGM, n (%) | 213 (48.1) |
|  | rtCGM, n (%) | 80 (18.1) |
|  | Insulin administration and glucose monitoring |  |
|  | MDI and SMPG, n (%) | 144 (32.5) |
|  | MDI and isCGM, n (%) | 203 (45.8) |
|  | MDI and rtCGM, n (%) | 23 (5.2) |
|  | CSII and SMPG, n (%) | 6 (1.4) |
|  | CSII and isCGM, n (%) | 10 (2.6) |
|  | CSII and rtCGM, n (%) | 57 (12.9) |
|  | Insulin regimen in MDI treatment (n=370) |  |
|  | Basal only, n (%) | 13 (3.5) |
|  | Basal bolus, n (%) | 357 (96.5) |
| **Biochemical and hormonal parameters** | | |
|  | HbA1c, % | 7.4 (6.7–7.9) |
|  | HbA1c, mmol/mol | 57.0 (50.0–63.0) |
|  | Creatinine, mg/dL | 0.82 (0.72–0.91) |
|  | eGFR, mL/(min ⋅ 1.73 m^2^) | 95.1 (82.0–108.0) |
|  | Albuminuria, mg/dL | 5.0 (5.0–8.0) |
|  | Total Cholesterol, mg/dL | 184.0 (160.0–205.0) |
|  | HDL Cholesterol, mg/dL | 61.0 (51.0–71.0) |
|  | Triglycerides, mg/dL | 70.0 (55.0–94.0) |
|  | LDL Cholesterol, mg/dL | 102.4 (89.0–120.2) |
|  | ALT, U/L | 18.0 (14.0-26.0) |
|  | AST, U/L | 22.0 (18.0–26.0) |
|  | TSH, μUI/mL | 1.93 (1.39–3.35) |
| **Associated diseases and complications** | | |
|  | Psychiatric disease*, n (%) | 29 (6.5) |
|  | History of cancer, n (%) | 17 (3.8) |
|  | Thyroid Disease, n (%) | 137 (30.9) |
|  | Autoimmune Thyroiditis, n (%) | 110 (24.8) |
|  | Celiac Disease, n (%) | 28 (6.3) |
|  | Patients with at least one additional autoimmune disorder, n (%) | 144 (32.5) |
|  | Hypertension, n (%) | 102 (23.0) |
|  | Cardiovascular Diseases, n (%) | 19 (4.3) |
|  | Diabetic Retinopathy, n (%) | 141 (31.8) |
|  | Diabetic Nephropathy, n (%) | 12 (2.7) |
| **PSQI data** | | |
|  | Altered sleep quality, n (%) | 142 (32.1) |
|  | Reduced sleep duration, n (%) | 177 (40.0) |
| **PSQI component scores** | | |
|  | Global score | 5.0 ± 3.09 |
|  | Component 1 (Sleep Quality) | 0.92 ± 0.66 |
|  | Component 2 (Sleep Latency) | 0.84 ± 0.86 |
|  | Component 3 (Sleep Duration) | 0.78 ± 0.74 |
|  | Component 4 (Habitual Sleep Efficiency) | 0.47 ± 0.82 |
|  | Component 5 (Sleep Disturbance) | 1.19 ± 0.50 |
|  | Component 6 (Use of Sleeping Medication) | 0.19 ± 0.66 |
|  | Component 7 (Daytime Dysfunction) | 0.61 ± 0.64 |
| Data are expressed as median with interquartile range in parentheses, or as frequencies. PSQI component scores are expressed as mean ± standard deviation.  T1D, type 1 diabetes; LADA, latent autoimmune diabetes of the adult; TDD, total daily (insulin) dose; MDI, multiple daily injections; CSII, continuous subcutaneous insulin infusion; SMPG, self-monitoring of plasma glucose; isCGM, intermittently scanned continuous glucose monitoring (device); rtCGM, real-time continuous glucose monitoring (device); HbA1c, glycated hemoglobin; eGFR, estimated Glomerular Filtration Rate (CKD-EPI); HDL, high-density lipoprotein; LDL, low-density lipoprotein; AST, aspartate aminotransferase; ALT, alanine aminotransferase; TSH, thyroid-stimulating hormone; PSQI, Pittsburgh Sleep Quality Index  * Psychiatric diseases included anxiety/depression (n=23), panic disorder (n=2), bipolar disorder (n=2), and eating disorders (n=2). | | |

**Supplementary Table 2. Population characteristics stratified by sleep duration (7 hours cut-off).**

|  | | Normal Sleepers  (n=266) | Short Sleepers  (n=177) | P value |
| --- | --- | --- | --- | --- |
| **Demographic and anthropometric characteristics** | | | | |
|  | Age, years | 46.0 (31.0-58.25) | 51.0 (37.50-59.0) | 0.067 |
|  | Sex: Female, n (%) | 131 (49.2) | 78 (44.1) | 0.285 |
|  | Body Mass Index, Kg/m^2^ | 24.0 (22.1-26.8) | 24.5 (22.1-27.1) | 0.690 |
|  | Smoker status |  |  |  |
|  | Non-smokers, n (%) | 158 (59.4) | 92 (52.0) | 0.292 |
|  | Actively smoking, n (%) | 55 (20.7) | 45 (25.4) |  |
|  | Previously smoking, n (%) | 53 (19.9) | 40 (22.6) |  |
| **Diabetes-related characteristics** | | | | |
|  | Diabetes Duration, years | 22.29 (12.4-31.29) | 21.79 (13.43-33.79) | 0.632 |
|  | Diagnosis |  |  |  |
|  | T1D, n (%) | 248 (93.2) | 166 (93.8) | 0.818 |
|  | LADA, n (%) | 18 (6.8) | 11 (6.2) |  |
|  | TDD/Kg Ratio, U/(day ⋅ Kg) | 0.54 (0.43-0.68) | 0.54 (0.40-0.69) | 0.826 |
|  | Insulin administration |  |  |  |
|  | MDI, n (%) | 224 (84.2) | 146 (82.5) | 0.632 |
|  | CSII, n (%) | 42 (15.8) | 31 (17.5) |  |
|  | Glucose monitoring: CGM |  |  |  |
|  | Glucose monitoring: device class |  |  |  |
|  | SMPG, n (%) | 92 (34.6) | 58 (32.8) | 0.123 |
|  | isCGM, n (%) | 134 (50.4) | 79 (44.6) |  |
|  | rtCGM, n (%) | 40 (15.0) | 40 (22.6) |  |
|  | Insulin regimen |  |  |  |
|  | Basal only, n (%) | 9 (4.0) | 4 (2.7) | 0.514 |
|  | Basal bolus, n (%) | 215 (96.0) | 142 (97.3) |  |
|  | **HbA1c, %** | **7.3 (6.6-7.8)** | **7.5 (6.9-8.1)** | **0.008** |
|  | **HbA1c, mmol/mol** | **56.0 (49.0-62.0)** | **58.0 (52.0-64.5)** |  |
| **Associated diseases and complications** | | | | |
|  | Psychiatric disease, n (%) | 15 (5.6) | 14 (7.9) | 0.344 |
|  | History of cancer, n (%) | 9 (3.4) | 8 (4.5) | 0.542 |
|  | Thyroid Disease, n (%) | 76 (28.6) | 61 (34.5) | 0.189 |
|  | Autoimmune Thyroiditis, n (%) | 59 (22.2) | 51 (28.8) | 0.113 |
|  | Celiac Disease, n (%) | 15 (5.6) | 13 (7.3) | 0.470 |
|  | Patients with at least one additional autoimmune disorder, n (%) | 79 (29.7) | 65 (36.7) | 0.122 |
|  | Hypertension, n (%) | 58 (21.8) | 44 (24.9) | 0.455 |
|  | Cardiovascular Diseases, n (%) | 9 (3.4) | 10 (5.6) | 0.249 |
|  | Diabetic Retinopathy, n (%) | 86 (32.3) | 55 (31.1) | 0.781 |
|  | Diabetic Nephropathy, n (%) | 8 (3.0) | 4 (2.3) | 0.635 |
| Data are expressed as median with interquartile range in parentheses, or as frequencies.  T1D, type 1 diabetes; LADA, latent autoimmune diabetes of the adult; TDD, total daily (insulin) dose; MDI, multiple daily injections; CSII, continuous subcutaneous insulin infusion; SMPG, self-monitoring of plasma glucose; isCGM, intermittently scanned continuous glucose monitoring (device); rtCGM, real-time continuous glucose monitoring (device); HbA1c, glycated hemoglobin. | | | | |

**Supplementary Table 3. Questionnaire scores by sleep quality.**

|  | Males | | | | Females | | |
| --- | --- | --- | --- | --- | --- | --- | --- |
|  | **Normal Sleepers** | **Poor Sleepers** | **P value** | **Normal Sleepers** | | **Poor Sleepers** | **P value** |
| **Pittsburgh Sleep Quality Index** | | | | | | | |
| Population, n | 166 | 68 |  | 135 | | 74 |  |
| **Global score** | **3.24 (±1.31)** | **8.15 (±2.59)** | **<0.001** | **3.36 (±1.29)** | | **8.97 (±2.73)** | **<0.001** |
| **Component 1 (Sleep Quality)** | **0.70 (±0.47)** | **1.41 (±0.76)** | **<0.001** | **0.66 (±0.52)** | | **1.43 (±0.64)** | **<0.001** |
| **Component 2 (Sleep Latency)** | **0.44 (±0.60)** | **1.43 (±0.85)** | **<0.001** | **0.53 (±0.59)** | | **1.76 (±0.82)** | **<0.001** |
| **Component 3 (Sleep Duration)** | **0.60 (±0.55)** | **1.38 (±0.73)** | **<0.001** | **0.43 (±0.53)** | | **1.24 (±0.90)** | **<0.001** |
| **Component 4 (Habitual Sleep Efficiency)** | **0.10 (±0.33)** | **1.32 (±1.04)** | **<0.001** | **0.13 (±0.34)** | | **1.11 (±1.00)** | **<0.001** |
| **Component 5 (Sleep Disturbance)** | **1.0 (±0.35)** | **1.37 (±0.51)** | **<0.001** | **1.07 (±0.35)** | | **1.66 (±0.63)** | **<0.001** |
| **Component 6 (Use of Sleeping Medication)** | **0.02 (±0.19)** | **0.40 (±0.98)** | **<0.001** | **0.01 (±0.09)** | | **0.68 (±1.14)** | **<0.001** |
| **Component 7 (Daytime Dysfunction)** | **0.38 (±0.52)** | **0.84 (±0.64)** | **<0.001** | **0.53 (±0.61)** | | **1.09 (±0.62)** | **<0.001** |
| **Diabetes Distress Scale** | | | | | | | |
| Population, n | 158 | 65 |  | 129 | | 68 |  |
| **Global score** | **1.47 (1.24-2.11)** | **2.0 (1.56-2.97)** | **<0.001** | **1.71 (1.29-2.26)** | | **2.41 (1.47-3.5)** | **0.002** |
| **Emotional burden score** | **1.8 (1.35-2.8)** | **2.4 (1.7-3.4)** | **0.004** | **2.0 (1.4-2.8)** | | **2.8 (1.85-4.15)** | **<0.001** |
| Physician-related distress score | 1.0 (1.0-1.75) | 1.25 (1.0-2.5) | 0.122 | 1.25 (1.0-2.25) | | 1.38 (1.00-2.75) | 0.286 |
| **Regimen-related distress score** | **1.6 (1.2-2.0)** | **2.0 (1.6-2.9)** | **<0.001** | **1.6 (1.2-2.3)** | | **2.0 (1.4-3.4)** | **0.002** |
| **Diabetes-related interpersonal distress score** | **1.33 (1.0-1.67)** | **1.67 (1.0-2.83)** | **<0.001** | **1.33 (1.0-2.17)** | | **2.0 (1.0-3.67)** | **0.010** |
| **Altered scores** |  |  |  |  | |  |  |
| **Global score, n (%)** | **45 (28.5)** | **32 (49.2)** | **0.003** | **43 (33.3)** | | **38 (55.9)** | **0.002** |
| **Emotional burden, n (%)** | **60 (38.0)** | **37 (56.9)** | **0.009** | **59 (45.7)** | | **46 (67.6)** | **0.003** |
| **Physician-related distress, n (%)** | **29 (18.4)** | **21 (32.3)** | **0.023** | 34 (26.4) | | 23 (33.8) | 0.272 |
| **Regimen-related distress, n (%)** | **35 (22.2)** | **32 (49.2)** | **<0.001** | **39 (30.2)** | | **32 (47.1)** | **0.019** |
| **Diabetes-related interpersonal distress, n (%)** | **22 (13.9)** | **24 (36.9)** | **<0.001** | **32 (24.8)** | | **29 (42.6)** | **0.010** |
| **Diabetes-related Quality of Life** | | | | | | | |
| Population, n | 120 | 45 |  | 96 | | 44 |  |
| **Score** | **1.62 (1.47-1.82)** | **1.98 (1.66-2.13)** | **<0.001** | **1.72 (1.56-1.91)** | | **2.09 (1.79-2.34)** | **<0.001** |
| **Diabetes Treatment Satisfaction Questionnaire status version** | | | | | | | |
| Population, n | 157 | 65 |  | 125 | | 69 |  |
| **Score** | **30.0 (26.0-34.0)** | **29.0 (25.0-31.5)** | **<0.001** | **31.0 (26.0-33.0)** | | **28.0 (23.5-32.0)** | **0.006** |
| Data are expressed as median with interquartile range in parentheses, or as frequencies. | | | | | | | |

**Supplementary Table 4. Continuous Glucose Monitoring indices in patients divided by sleep quality.**

|  | Normal Sleepers | Poor Sleepers | P value |
| --- | --- | --- | --- |
| Population, n | 68 | 28 |  |
| Glucose Management Indicator, % | 7.1 (6.8-7.6) | 7.2 (6.8-8.0) | 0.306 |
| Glucose Management Indicator, mmol/mol | 54.3 (50.4-59.5) | 55.6 (50.9-64.1) |  |
| Standard Deviation, mmol/mol | 60.5 (51.1-66.7) | 60.0 (50.3-69.9) | 0.678 |
| Coefficient of Variation, % | 36.8 (33.0-41.3) | 36.9 (31.9-40.9) | 0.693 |
| Time In Range (70-180 mg/dL), % | 60.0 (50.4-68.7) | 57.0 (43.5-70.7) | 0.362 |
| Time Below Range, level 1 (54-69 mg/dL), % | 2.9 (1.3-5.3) | 1.8 (0.8-4.8) | 0.182 |
| Time Below Range, level 2 (<54 mg/dL), % | 0.3 (0.8-1-1) | 0.2 (0.4-0.9) | 0.493 |
| Time Above Range, level 1 (180-250 mg/dL), % | 24.7 (18.8-29.0) | 25.9 (20.2-30.5) | 0.554 |
| Time Above Range, level 2 (>250 mg/dL), % | 8.6 (3.2-15.9) | 10.3 (3.0-19.2) | 0.388 |
| Data are expressed as median with interquartile range in parentheses. | | | |

**Supplementary Figure 1. Flow chart of the patients’ selection process.**

Enrolled subjects (n=533)

Excluded for PSQI compilation error (n=44)

Excluded for missing clinical data (n=44)

Analyzed subjects (n=443)

Excluded for missing HbA1c (n=22)
